# Identification of Motor and Mental Imagery EEG in Two and Multiclass Subject-Dependent Tasks Using Successive Decomposition Index

**DOI:** 10.3390/s20185283

**Published:** 2020-09-16

**Authors:** Muhammad Tariq Sadiq, Xiaojun Yu, Zhaohui Yuan, Muhammad Zulkifal Aziz

**Affiliations:** School of Automation, Northwestern Polytechnical University, 127 West Youyi Road, Xi’an 710072, China; tariq.sadiq@mail.nwpu.edu.cn (M.T.S.); XJYU@nwpu.edu.cn (X.Y.); zulkifalaziz@mail.nwpu.edu.cn (M.Z.A.)

**Keywords:** electroencephalography, Brain-Computer Interface, multiscale principal component analysis, successive decomposition index, motor imagery, mental imagery, neurorehabilitation, classification

## Abstract

The development of fast and robust brain–computer interface (BCI) systems requires non-complex and efficient computational tools. The modern procedures adopted for this purpose are complex which limits their use in practical applications. In this study, for the first time, and to the best of our knowledge, a successive decomposition index (SDI)-based feature extraction approach is utilized for the classification of motor and mental imagery electroencephalography (EEG) tasks. First of all, the public datasets IVa, IVb, and V from BCI competition III were denoised using multiscale principal analysis (MSPCA), and then a SDI feature was calculated corresponding to each trial of the data. Finally, six benchmark machine learning and neural network classifiers were used to evaluate the performance of the proposed method. All the experiments were performed for motor and mental imagery datasets in binary and multiclass applications using a 10-fold cross-validation method. Furthermore, computerized automatic detection of motor and mental imagery using SDI (CADMMI-SDI) is developed to describe the proposed approach practically. The experimental results suggest that the highest classification accuracy of 97.46% (Dataset IVa), 99.52% (Dataset IVb), and 99.33% (Dataset V) was obtained using feedforward neural network classifier. Moreover, a series of experiments, namely, statistical analysis, channels variation, classifier parameters variation, processed and unprocessed data, and computational complexity, were performed and it was concluded that SDI is robust for noise, and a non-complex and efficient biomarker for the development of fast and accurate motor and mental imagery BCI systems.

## 1. Introduction

With the rampant growth in automated systems, computer-aided physical systems, and artificial intelligence, brain–computer interface (BCI) has gained significant attention from researchers as it can bind a human mind to the computer and operate complex physical applications. The healthcare realm has been overwhelmed by the development of computer-aided brain devices, namely, prosthetic arms, brain-controlled wheelchairs, mind-controlled home automation, etc., for physically impaired people [1,2,3,4,5,6,7]. The fundamental source of BCI is the low-key signal generated on the surface of the human scalp as a result of neural activity and it acts as a watershed for the plethora of brain-controlled applications.

The common practices involved to retrieve such signals are invasive and noninvasive methods. Invasive methods, as the name implies, record signals from the inside of the human brain which results in artifact-free data. On the other hand, noninvasive techniques accumulate noise artifacts that degrade the performance of BCI systems. Electroencephalography (EEG) is the commonly employed technology for the development of practical BCI systems.

Motor and mental imagery are subdomains in BCI which deal with the simulation of motor and mental activities in the brain without performing any activity in real. The inherent nature of motor and mental imagery suggests an economical, noninvasive, portable, and high temporal resolution mode of acquiring signals and the best choice is electroencephalography (EEG) [3,8]. After acquiring the signal, the subsequent process is to correctly wring out useful information from it [9,10].

The analysis of any signal processing problem comprises at least three basic procedures: preprocessing (data preparation and artifacts removing), feature extraction (identifies the most significant characteristics in signals), and classification (segregating classes between features). As noninvasive mode of signal acquisition heavily accumulates noise artifacts and it is crucial to filter out alienated signals without disturbing the original content. Recent studies proposed independent component analysis (ICA) [11], principal component analysis, and canonical correlation analysis [12] for the noise removal of EEG signals; however, these methods are not very effective for the analysis of non-stationary signals [12]. Another hybrid algorithm namely multiscale principal component analysis (MSPCA) is recently proposed and studies [13,14] revealed its robustness in denoising non-stationary and nonlinear signals.

After preprocessing the data, the subsequent steps are features estimation and classification [15]. In EEG signal processing, some widely adopted feature extraction methods are categorized as Fourier transform (FT) [16], power spectral density (PSD) [17], common spatial patterns (CSP) [18,19], autoregressive (AR) [20,21], sparse representation, and signal decomposition (SD) [22,23,24] based methods. All of these methods have their associated demerits and complications, for example, FT-based features only preserve the spectral resolution of the signal and completely loses the temporal information, PSD-based methods are susceptible to electrodes locality, AR-based techniques are sensitive to noise content, etc.

Chattarjee et al. [25] does a comparative analysis for a different time, energy, entropy, and statistical features using a different machine and deep learning classifiers for EEG signals. The maximum classification outcome of 85% was observed for energy and entropy-based features using the support vector machine (SVM) classifier. Wang et al. [26] amalgamates empirical mode decomposition (EMD) with Hilbert spectral analysis for motor imagery EEG signals and backpropagation neural network for classification purposes. The maximum recorded accuracy was 93.8%. Gupta et al. [17] extracted PSD features for EEG signals and did a comparative analysis for different univariate and multivariate features selection methods using different classifiers. A maximum classification accuracy of 85% was obtained for the combination of the Burg and linear regression features selection method using the linear discriminant analysis classifier.

Jasmine et al. [22] presents a comparative analysis for three signal decomposition techniques, i.e., EMD, discrete wavelet transform, and wavelet packet decomposition (WPD), using motor imagery EEG datasets. The highest accuracy of 92.8% was attained for higher-order statistical features extracted from WPD using K-nearest neighbors classifier. Chaudhary et al. [27] combines the non-dyadic wavelet decomposition method and CSP features extraction method for the classification of motor imagery EEG signals. Maximum classification accuracy of 85.6% was obtained for decision tree classifier. Jiacan et al. [28] presents a deep multi-view feature learning process for the classification of motor imagery EEG tasks. First, many multidomain features (time, frequency, time-frequency, and spatial) were extracted, and then a restricted Boltzmann machine network improved by t-distributed stochastic neighbor embedding (t-SNE) is employed for features learning. An average classification accuracy of 78.5% was obtained using the SVM classifier. Chen et al. [29] develops an NAO robot walking control system based on motor imagery by utilizing CSP and local characteristic scale decomposition (LCD). The experimental results yielded a classification accuracy of 87.5%.

Our study [23] proposed an instantaneous amplitude and instantaneous frequency component-based features. First, the empirical wavelet transform (EWT) was employed to decompose an EEG signal into representative modes, then the Welch PSD method was adopted for modes selection. The last step was to calculate the instantaneous components of each selected mode and classify the features with seven machine learning classifiers. The maximum accuracy achieved was 95.2% for the proposed mechanism. Our second study [24] on motor imagery EEG proposed a multivariate empirical wavelet transform (MEWT) for signal decomposition. By selecting features with correlation-based method and classifying them with three benchmark classifiers, we obtained 98% classification outcomes for the least square version of SVM classifier. All the methods discussed above, either utilized complex signal decomposition methods in combination with features selection methods or used complex features extraction methods, which are both impractical for the realization of functional BCI system. Raghu et al. [30] proposed the successive decomposition index (SDI) method for the classification of epileptic seizures. The classification outcomes suggested that SDI is a successful feature extraction method for epileptic seizures and it can be extended to other EEG domains.

Many different studies have built graphical user interface (GUI) systems for the visual implementation of their proposed approaches. EPILAB GUI was developed by Teixeirra et al. [31] for the analysis and classification of epileptic seizures. EEGLAB developed by Delorme et al. [32] presented an ICA-based EEG signal denoising method, time-frequency analysis, and visual representation of EEG signals. Moreover, Oostenveld et al. [33] reviews a MATLAB open source toolbox named FieldTrip, which does the time-frequency analysis, non-parametrical statistical tests, and reconstruction using dipoles and distributed sources of EEG and magnetoencephalography (MEG) signals. Each of these methods analyzes multidomain EEG signals, but a specialized GUI for motor and mental imagery is lagging.

For the robust, efficient, and non-complex analysis and classification of motor and mental imagery EEG signals, this article for the first time to the best of our knowledge and understanding, makes use of successive decomposition index (SDI) for feature extraction. This research attests the performance of SDI feature using six benchmark machine learning and neural network classifiers and different case studies confirms the effectiveness of proposed method. The main contributions of this study are listed as follows:

1. Successive decomposition index is proposed for the decoding of different motor and mental imagery activities in development of the BCI system.
2. Statistical analysis and novel performance evaluation criteria named polygon area metrics (PAM) are performed to confirm the efficacy of the SDI feature as a biomarker.
3. Four different channel selection schemes are employed to validate the performance of SDI features corresponding to the number of channels.
4. Classifier parameters are varied to investigate its fallouts on the proposed method.
5. A comparison was undertaken for denoised and noisy data to confirm the robustness of SDI features against noise artifacts.
6. Validate the performance of the proposed approach for multiclass mental imagery data.
7. Developed a computerized automatic detection of motor and mental imagery-successive decomposition index (CADMMI-SDI) application for the visual and practical implementation of SDI features.

The rest of paper is organized as follows. Section 2 and Section 3 deal with the datasets and the description of methods employed during the study, Section 4 describes the performance measures, Section 5 presents experimental set-up, Section 6 provides the results and discussion of the experimental outcomes, and, finally, Section 7 summarizes the study.

## 2. Materials

This study makes use of three motor and mental imagery publicly available datasets: IVa, IVb, and V from BCI competition III. Dataset IVa is a motor imagery dataset with two tasks right hand (RH) (Class 1) and right foot (RF) (Class 2). Five normal subjects or participants (“aa”, “al”, “av”, “aw”, and “ay”) participated for the collection of datasets. The global 10-20 system was used for the placement of 118 electrodes on the scalp. All the participants were shown a visual sign for 3.5 s and a total number of 280 trials (140 trials for each class) were recorded for an individual participant and the data were sampled at 1000 Hz. Similarly, dataset IVb is another single participant binary class motor imagery dataset with tasks left hand (LH) (Class 1) and right foot (RF) (Class 2). The data acquisition parameters for dataset IVb are similar to dataset IVa. Dataset V is a data collection of 3 individuals with imaginative roles of LH movement, RH movement, and random word (RW) production. These tasks are named as Class 1, Class 2, and Class 3, respectively. Data was collected in three cycles from 3 individuals with 32 electrodes and sampling frequency of 512 Hz. Further information for data sets is presented online at http://www.bbci.de/competition/iii/.

## 3. Methods

**Figure 1:**
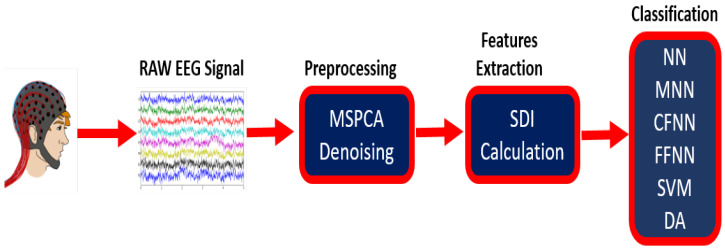
Block diagram of the successive decomposition index for identification of motor and mental imagery activities.

The study proposed a SDI-based framework for automated classification of two and multi-category motor and mental imagery EEG tasks in the development of computer-aided BCI systems. Figure 1 shows a clear presentation of the proposed strategy. First, the MSPCA process is used to separate noise from the raw EEG signal. Afterward, SDI is employed, that is, an inspirational case of discrete wavelet transform where a time series is pass through *n* levels of low-pass and high-pass filters and the coefficient at each step is used as a feature, and at last the extracted features are used as the inputs to the several machine leaning and neural network classifiers. Moreover, this study built up a layout for the realistic implementation of proposed platform for identifying motor and mental imagery EEG signals known as computerized automated detection of motor and mental imagery successive decomposition index (CADMMI-SDI). The subsequent subsections describe the details of the proposed automated framework.

### 3.1. Module 1: MSPCA Denoising

**Figure 2:**
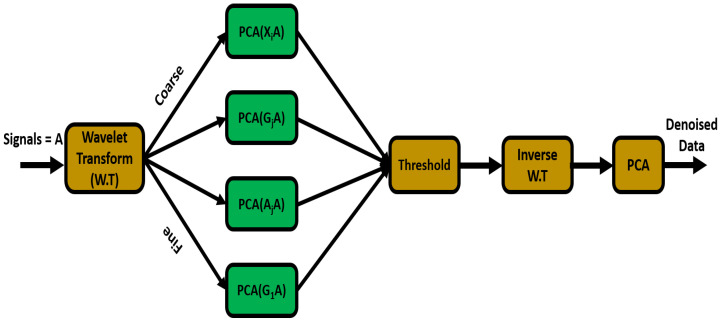
Multiscale principal component analysis (MSPCA) for denoising.

EEG is a noninvasive method of signal retrieval from the subject that inherits different types of noise artifacts, i.e., systematic noise, blink signal noise, cardiac signals noise, thermal noise, etc. A mathematical model of the crude form signal can be described as follows [34],

(1) $$X=X_{EEG}+X_{N}$$

where $X_{EEG}$ is the desired EEG signal and $X_{N}$ is the supplemental noise artifact added to the original signal. The objective is to model a system that can effectively remove noise from the raw signal without influencing the content of $X_{EEG}$. Principal Component Analysis (PCA) is conventionally adopted for determining the linear relationship between correlated data points. Furthermore, the nonlinear and non-stationary nature of the EEG signal demands a time-frequency resolution. Therefore, wavelet transform is commonly adopted and its significance is widely tested for non-stationary and nonlinear signals. A hybrid signal denoising algorithm called multiscale principal component analysis (MSPCA) is formulated by combining the properties of PCA and wavelet transform [24]. The workflow of MSPCA is given in Figure 2. We can define the procedure as follows.

1. Take a matrix *A* with dimensions n×m, where *n* is the length of each signal and *m* is the number of channels. Decompose each channel into *B* levels using wavelet transform.
2. Formulate a detailed matrix $A_{j}A$ and approximation matrix $X_{i}A$, and calculate PCA for all *B* decompositions and *m* channels. As the Kaiser rule suggests, select principal components with eigenvalues greater than the mean of collective eigenvalues.
3. Compute the inverse wavelet transform of the selected principal components.
4. A denoised signals matrix can be obtained by taking the PCA of the results obtained in step 3.

### 3.2. Module 2: Successive Decomposition Index Based Feature Extraction

In the past, a large number of studies [22,24,35,36] investigated the effectiveness of wavelet and signal decomposition-based methods for motor and mental imagery EEG signals using different mother wavelets and decomposition levels. The drawbacks of such methods are the selection of suitable mother wavelets and the number of decomposition levels which requires a thorough investigation in terms of classification outcomes and time complexity. The basic requirements of a practical BCI system are robustness, non-complexity and efficiency that are lagging in current researches. To overcome the aforementioned limitations a successive decomposition index (SDI) method is employed.

The proposed SDI method is an inspiration of discrete wavelet transform (DWT). In the first level of DWT, a time signal of length *n* is passed through a low and high pass filter. In the next level, the output of low pass filter is again passed through a high and low pass filter and this process is iterated for a specific number of decomposition levels. Finally, the coefficients from each decomposition level are used to extract features. The basic difference between DWT and SDI is that the former has to have a predefined number of decomposition levels whether the later has no predefined decomposition levels and the coefficient from the last level is considered for further analysis. The mathematical formulation of the SDI feature is described in following steps [30].

1. Consider an EEG signal $s=\{s_{1},s_{2},s_{3},\ldots \ldots \ldots .,s_{n}\}$, where *n* is the length of the signal. The first step is to compute the average of absolute values ($S^{+}$ ) of the EEG signal is as follows.
  (2) $$S^{+}=\frac{1}{n}\sum_{i=1}^{n}\left|s_{i}\right|$$
2. The next step is to compute the average difference ($S^{-}$) of the signal and it can be calculated by the successive difference mean of non-overlapping pairs of time signal. It can mathematically represented as follows,
  (3) $$s^{\left(1\right)}=\left\{\frac{s_{1}-s_{2}}{2},\frac{s_{3}-s_{4}}{2}\cdots \cdots ,\frac{s_{n-3}-s_{n-2}}{2},\frac{s_{n-1}-s_{n}}{2}\right\}$$
  where the length of $s^{\left(1\right)}$ is n/2. Similarly, $s^{\left(2\right)}$ can be calculated as
  (4) $$s^{\left(2\right)}=\left\{\frac{s_{1}^{\left(1\right)}-s_{2}^{\left(1\right)}}{2},\frac{s_{3}^{\left(1\right)}-s_{4}^{\left(1\right)}}{2}\cdots \cdots ,\frac{s_{n/2-3}^{\left(1\right)}-s_{n/2-2}^{\left(1\right)}}{2},\frac{s_{n/2-1}^{\left(1\right)}-s_{n/2}^{\left(1\right)}}{2}\right\}$$
  The process of calculating s(k) (where *k* is the number of iterations) continues until we get a single coefficient and that final coefficient is the average difference term $S^{-}$. The number of iterations required to calculate $S^{-}$ can be determined as k=3.33log10(n) and the total number of coefficients at each step are $n/2^{k}$. The next step is to calculate two new terms $S^{++}$ and $S^{--}$ as follows.
  (5) $$S^{++}=\frac{S^{+}+S^{-}}{2}$$
  (6) $$S^{--}=\frac{S^{+}-S^{-}}{2}$$
  The terms $S^{++}$ and $S^{--}$ gives the relation between $S^{+}$ and $S^{-}$. In addition, a square matrix *Z* is formed from the four coefficients as follows.
  (7) $$Z=\left[\begin{array}{cc} S^{+} & S^{--}\\ S^{-} & S^{++} \end{array}\right]$$
3. The final step is to calculate the determinant of matrix *Z* multiplied by a scalar n/k followed by log10.
  (8) $$SDI=\log_{10}\left(\frac{n}{k}\left(S^{+}S^{++}-S^{-}S^{--}\right)\right)$$

The resultant SDI is a single value bio marker for an EEG signal of length *n*. The significance of SDI is that it measures the variations of EEG signal successively with respect of time and packs it into a single representative value. In addition, unlike other wavelet and signal decomposition-based methods, there is no need to select a suitable mother wavelet and define the number of decomposition levels rather the process of calculating SDI is linear and non-complex, which makes it a suitable choice for the development of practical motor and mental imagery BCI systems.

### 3.3. Module 3: Classification

To segregate the motor and metal imagery tasks, we have utilized six widely used machine learning and neural network classifiers. Their description and parameters of classifiers utilized in this study are discussed as follows.

#### 3.3.1. Support Vector Machine

A support vector machine (SVM) is a supervised learning classifier that formulates a hyperplane to maximize the separability between two classes. For nonlinear feature sets, different kernel functions are utilized to transform it into a linear problem at the cost of augmented dimensionality. The selection of SVM in this study is based on its robustness and reliability for motor imagery tasks discussed in [37,38]. In this study, we have utilized the radial basis function, linear function, and polynomial function as kernels and the default MATLAB toolbox hyperparameters were availed for each kernel.

#### 3.3.2. Discriminant Analysis

Discriminative analysis (DA) is a supervised learning algorithm that formulates a predictive model during the learning phase that can be applied to test data for labeling them. DA can use lines, planes, and hyperplanes to segregate the normally distributed samples and thus it can classify multidimensional data robustly. To build a DA model, we have to compute the class probability, mean, and covariance matrix along with a suitable kernel function. In this study, we have utilized three kernels: linear, pseudo-linear, and pseudo-quadratic. The effectiveness of DA for motor imagery tasks has been accredited in [39,40].

#### 3.3.3. Multilayer Perceptron with One Hidden Layers

A multilayer perceptron with single hidden layer (ANN) is the building block of deep learning classifiers and is robust in approximating linear, nonlinear functions and pattern recognition effectively. ANN has a three-layered structure consisting of input, hidden, and output layers. The number of input nodes is same as the number of features while the number of output nodes is equal to the number of classes. The number of hidden nodes is variable and depends primarily on classification outcomes. ANN propagates the input signal from first to last layer and the backpropagation algorithm tunes the hyperparameters of the network during training phase. The studies [41,42] attests the robustness of ANN for motor imagery EEG.

#### 3.3.4. Multilayer Perceptron with Two Hidden Layers

A multilayer perceptron with two hidden layers (MNN) is an extension of ANN. The basic difference between both algorithms is that MNN has 2 to *M* hidden layers depending upon the classification results while ANN has only one hidden layer. The advantage of using MNN is that it has more parameters and hence it has an extra degree of freedom to approximate a nonlinear function or recognize a pattern. The disadvantage is that, because of the large number of hyperparameters, the training and testing time exceeds ANN and hence there is a trade-off between computational time and classification outcomes.

#### 3.3.5. Cascade Feedforward Neural Network

The architecture of cascade feedforward neural network (CFNN) resembles ANN. The core difference between both classifiers is that CFNN has a connection from the output layer to the input layer that ANN lags in its structure. This extra connection gives CFNN the ability to memorize previous inputs and their outcomes and thus it is essential in learning sequential data. The authors of [43] utilized CFNN for the classification of motor imagery tasks.

#### 3.3.6. Feed-Forward Neural Network

Throughout the feed-forward neural network (FFNN), a multilayered structure is used with each layer containing variable number of neurons. The signal is propagated from input to output across the network and an error is computed using a cost function. This error is then repropagated across the network and each parameter is tuned. In our research, tan sigmoid was used as an activation feature. The Levenberg–Marquardt algorithm was used for fast learning [43].

There is no structural difference between ANN and FFNN. In the present study, we utilized two different MATLAB functions named “patternenet()” for ANN and “feedforwardnet()” for FFNN. The basic difference between these two functions is that ANN uses “glorot” weights and biases initializer while FFNN uses “orthogonal” initializer. The “glorot” initializer takes random samples from a normal distribution where mean is zero and variance is 2/(size of inputs + size of outputs), while the orthogonal initializer takes a matrix from a unit uniform distribution and initializes the weights and biases with Q obtained from a QR decomposition [44,45].

## 4. Performance Parameters

This study utilizes a 10-fold cross-validation method to fairly evaluate the classification results. For this purpose, the feature matrix containing Class 1 and Class 2 features is divided into 10 equal parts, out of that 9 parts were used for training purposes and 1 part was used for validation. In this way, each trial of the feature set is being trained upon as well as validated. To evaluate the classification outcomes, we made use of 10-fold cross-validation method with different performance metrics, namely, classification accuracy (Acc), Sensitivity (Sen), Specificity (Spe), Kappa, and F1-Score. Their mathematical expressions are given respectively as follows,

(9) $$A_{cc}=\frac{TP+TN}{TP+TN+FP+FN}$$

(10) $$S_{en}=\frac{TP}{TP+FN}$$

(11) $$S_{pe}=\frac{TN}{TN+FP}$$

(12) $$Kappa=\frac{TP\times TN-FP\times FN}{\sqrt{\left((TP+FP)(TP+FN)(TN+FP)(TN+FN)\right)}}$$

(13) $$F1_{-}Score=2\times \frac{\mathrm{Prec}\times S_{en}}{\mathrm{Prec}+S_{en}}$$

where TP (True positive) is the amount of adequately identify Class 1 labels, TN (True negative) is the amount of adequately identify Class 2 labels, FP (False positive) is the number of inadequately classified Class 1 labels. and FN (False negative) is the number of inadequately identified Class 2 labels.

Apart from the above mentioned five performance parameters, we utilized a novel performance evaluation criteria named polygon area metrics (PAM) [46] for the very first time for motor and mental imagery EEG classification evaluation. The PAM constructs a hexagon with six performance parameters (F measure, Jaccard Index, Classification accuracy, Area under the curve, Sensitivity and Specificity) on each edge. The performance in this case is evaluated by the area of the polygon. The greater the area occupied by the polygon, the better the performance of the classifier and vice versa.

## 5. Experimental Setup

All experiments and simulations in this study were performed using MATLAB R2019b on an Intel(R) Core (TM) M-5Y10c CPU @0.80GHz cpu, Windows 10 64-bit operating system, and 8 GB RAM with WEKA 3.8.4.

Numerous studies have been performed in the past for effectively classifying motor and mental imagery tasks as detailed in Section 1. Most of them utilized complex signal processing techniques that make those unfeasible for the practical implementation and it also gets difficult for physicians to understand complex signal processing tools without having a piece of proper knowledge about the field. To cope up with such challenges, we have utilized a single non-complex feature that uses iterative signal decomposition coefficients to construct a representative feature with the least computational complexity and effective classification results.

Figure 1 shows the block diagram of the proposed methodology. At first, the raw data is passed through an MSPCA filter that suppresses the noise content from the signals. Then the data is divided into individual trials. In the case of dataset IVa and IVb, the single trial dimension is 400 × 118, where 400 is the signal length and 118 is the number of channels. For dataset V, the single-trial dimension is 512 × 32, where 512 is the signal length and 32 is the number of channels. Next, each trial is given to an SDI computational function which calculates features for that trial. In the case of dataset IVa and IVb, we get 118 features for a single trial while for dataset V, we get 32 features for a single trial. In this way, a features matrix is formed with dimensions n × m, where *n* is the number of trials and *m* is the number of features (indirectly the number of channels) per trial. Last, the feature matrices of various classes are given to six benchmark classifiers to evaluate the performance of SDI features in estimating motor and mental imagery tasks.

## 6. Results and Discussion

### 6.1. Statistical Analysis

**Figure 3:**
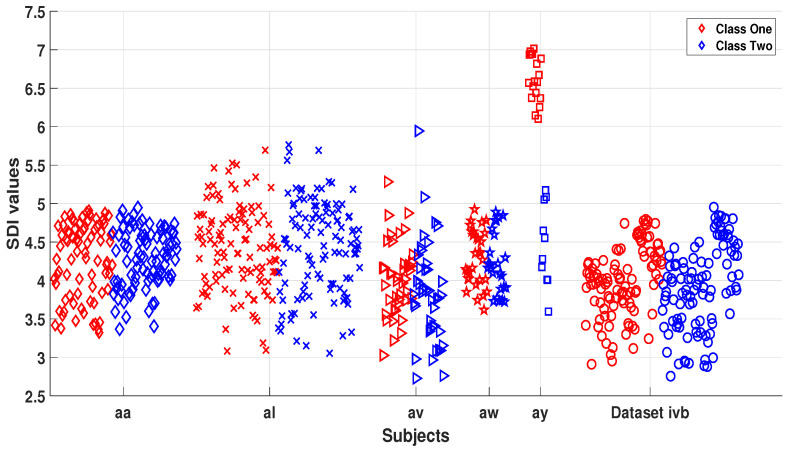
Scatter plot of SDI features for dataset IVa and IVb subjects.

To analyze how the SDI feature segregates motor imagery tasks, we have performed a statistical analysis in this section. Figure 3 presents the SDI feature distribution for Class 1 and Class 2 tasks by utilizing channel C3 from all subjects of dataset IVa and IVb. Figure 3 suggests that subjects “aa”, “al”, “av”, “aw”, “ay”, and dataset IVb have a highly nonlinear relationship between both task features and it is imperative to use a nonlinear classifier to trace the pattern between both classes. It can be seen in the Figure 3 that SDI feature has significantly singled out tasks for small training samples subject “ay” and later in this study we will see that subject “ay” is the best performant among all other subjects in terms of classification outcomes.

In addition, a descriptive statistical analysis in terms of mean, standard deviation, median, and Kruskal–Wallis probability (*p*) values (KW test) of SDI features was performed for single trial cases of each subject. The results presented in Table 1 suggest that the mean and median values of subject “aa”, “al”, “av”, “aw”, “ay”, and dataset IVb are higher for Class 2 cases than Class 1. For the subject “ay”, the mean and median values for Class 1 are higher and this trend was consistent for all trials. Moreover, the KW *p* values for single-trial cases of all subjects are less than 0.05 which suggests the significance of SDI features for motor imagery tasks and the high discrimination ability of extracted features between two classes.

### 6.2. Results by Selecting Different Number of Channels

**Figure 4:**
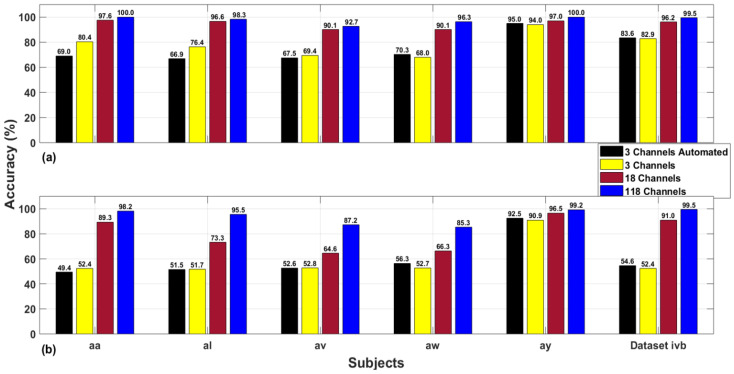
Bar plots for the comparison of 3-channel automated, 3-channel, 18-channel, and 118-channel results: (**a**) FFNN classifier and (**b**) SVM classifier.

Siuly et al. [47] conducted a comparative analysis for 18 and 118 channels motor imagery dataset IVa and IVb using two classification algorithms. Their study concludes that 118 channel results outperform 18 channels in terms of classification outcomes. In this section, a similar type of comparison is presented for dataset IVa and IVb with 18 channels, three channels and three channels selected with automated channel selection criteria. The 18 and three channels are widely adopted motor cortex channels while three-channel selection with automated channel selection criterion was proposed in our previous study [24]. The list of automated channels for each subject is given in Table 2. As motor imagery EEG signals are highly dependent upon subject physical and mental nature so for each subject, different channels are selected by the automated channel selection criteria. Figure 4 shows a visual representation of four channels selection schemes for best and worst-performing classifiers. The worst classifier is characterized in terms of least gain in accuracy while the best classifier symbolizes maximum gain in classification accuracy. This study made use of six machine learning and neural network classifiers (NN, MNN, CFNN, FFNN, SVM, and DA) out of which FFNN was the best performing classifier and SVM was the worst performer. The rest of the analysis is given as follows.

1. It is inferred from Figure 4 that 118 channels give the highest classification accuracy for dataset IVa as compared to other channel combinations. The average classification accuracy obtained using 118 channels with FFNN classifier is 97.46%. Similarly, the average accuracy for SVM classifier using 118 channels is 93.05%. Moving on to the 18-channel combination, it is observed that the average classification accuracy for FFNN and SVM classifier is 94.28% and 77.96% respectively. Furthermore, the 3-channel scheme resulted in a mean accuracy of 77.6% and 60.1% for FFNN and SVM classifiers respectively. Finally, the 3-channel automated scheme has the least average results as compared to other channels combination. The average results obtained for 3-channel automated criteria are 73.74% and 60.46% for FFNN and SVM classifiers, respectively.
2. For dataset IVb, a similar trend of classification accuracies is observed for varying channel combinations. Figure 4 shows that FFNN and SVM resulted in 99.5% accuracy each using 118 channels for dataset IVb and this is the highest among other channel selection schemes. For the combination of 18 channels, the FFNN classifier resulted in 96.2% accuracy and SVM yielded 91% classification certainty. Moving forward to the 3-channel strategy, it is noticed that FFNN turnout 82.9% accuracy and 52.4% for SVM. Likewise, 3-channel automated scheme resulted in similar pattern of results with 83.6% for FFNN and 54.6% for SVM classifier.
3. It is observed that the 118-channel combination has a maximum gain of 23.72% and 32.59% for FFNN and SVM classifiers, respectively using dataset IVa. Dataset IVb has a maximum gain of 16.6% and 47.1% for FFNN and SVM classifiers, respectively. It accredits the significance of using 118 channels for SDI features and advocates the channel comparison study performed by Siuly et al. [47].
4. One interesting observation is made that subject “ay” of dataset IVa has above 90% classification accuracy for all channel combinations and classifiers. As mentioned in the descriptive analysis section, the SDI features for subject “ay” tasks are well separated and distinguishable. We conclude that SDI feature extraction is more significant for subject with small training samples as compared to large one and this property makes it feasible for the development of practical BCI systems as disabled patients need small training to train a device.

### 6.3. Analysis with Sensitivity, Specificity, Kappa, F1-Score and PAM

**Figure 5:**
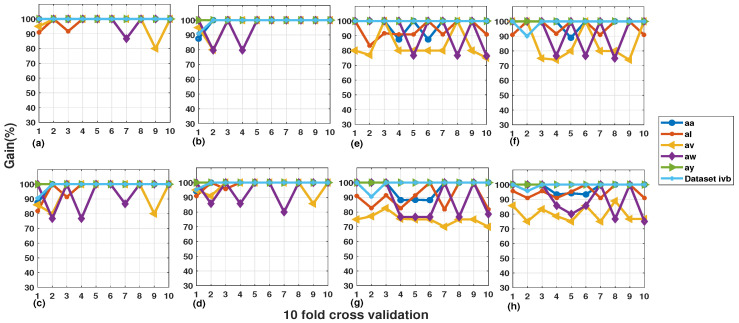
(**a**–**d**) 10-fold Sensitivity, Specificity, Kappa, F1-Score for FFNN Classifier. (**e**–**h**) Ten-fold Sensitivity, Specificity, Kappa, and F1-Score for SVM Classifier.

In this section, we explain the effect of other performance measures namely sensitivity, specificity, kappa, F1-Score and most importantly a unified novel performance measure, the polygon area metric (PAM). Figure 5 shows the sensitivity, specificity, kappa, and F1-score values for FFNN and SVM classifiers using 118 channels with 10-fold cross-validation strategy. Figure 5a,e show the sensitivity values for FFNN and SVM classifier, respectively. The average sensitivity values are 98.8% and 94.8% accordingly for individual classifiers which suggests that FFNN correctly identified Class 1 instances 98 times and SVM classified them correctly 94 times. Similarly, Figure 5b,f show the specificity values for FFNN and SVM classifiers respectively. The average 10-fold specificity values are 98.25% and 95.57%, respectively, for each classifier, which indicates that FFNN classified Class 2 instances effectively 98 times and SVM classified them positively 95 times. Figure 5c,g presents the kappa scores for the aforementioned classifiers. It is noted that the average kappa for FFNN classifier is 96.93% with slight variations for subject “aw”. The average kappa for SVM classifier is 91.5% with major variations in subject “av” and “aw”. Hence, we conclude that FFNN is more stable and unbiased in classifying Class 1 and Class 2 tasks. Finally, Figure 5d,h show the F1-Score for each classifier, respectively, and the average F1-Score for individual classifier is 98.07% and 93.83% accordingly. The high value of F1-Score for FFNN classifier illustrates the high precision and recall measures.

**Figure 6:**
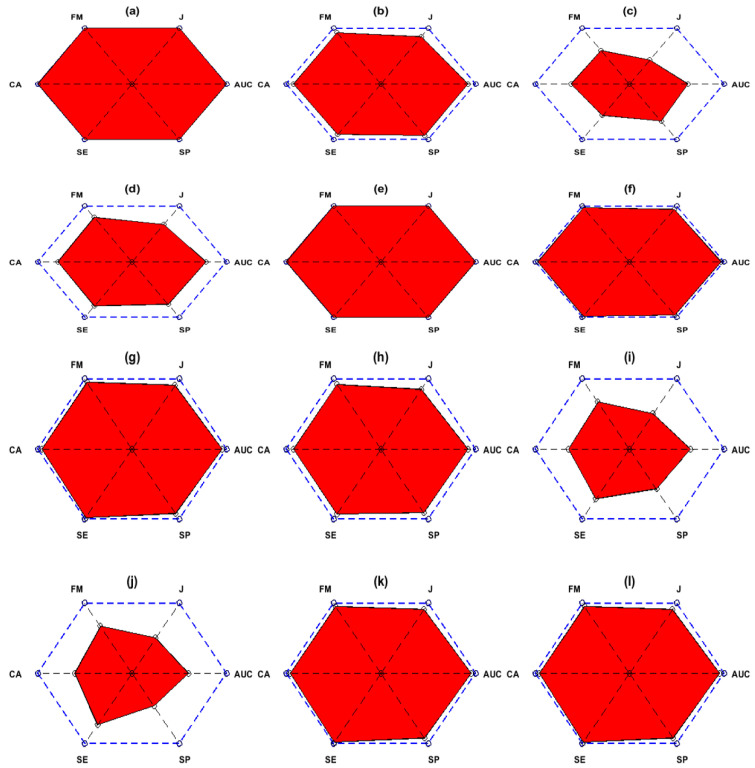
(**a**–**f**) PAM for Subjects “aa”, “al”, “av”, “aw”, “ay” and “Dataset IVb” respectively using FFNN classifier. (**g**–**l**) PAM for Subjects “aa”, “al”, “av”, “aw”, “ay” and “Dataset IVb” respectively for SVM classifier.

Figure 6 shows the PAM graphs for dataset IVa all subjects and dataset IVb using FFNN and SVM classifiers for 118 channels scheme. Figure 6a–f presents the PAM graphs for FFNN classifier and Figure 6g–l shows PAM graphs for SVM classifier. It can be seen that subject “aa” and “ay” have an area of 1 unit while subjects “al”, “av”, and “a” have areas of 0.95, 0.78, and 0.85 units for FFNN classifier, respectively. Dataset IVb has an area of 0.98 units for FFNN classifier. All of these results are consistent with the above-mentioned accuracy and other performance measures outcomes. Moreover, in the case of SVM classifier, subject “aa”, “ay” and dataset IVb has an area of 0.98 units each, subject “al”, “av”, “aw”, and “ay” has an area 0.95, 0.81, and 0.79 units, respectively. The key benefit of using PAM graph is that complete classification performance is represented in a single graph with several measures instead of looking into lengthy tables.

### 6.4. Results by Selecting Different Parameters of Classifiers

**Figure 7:**
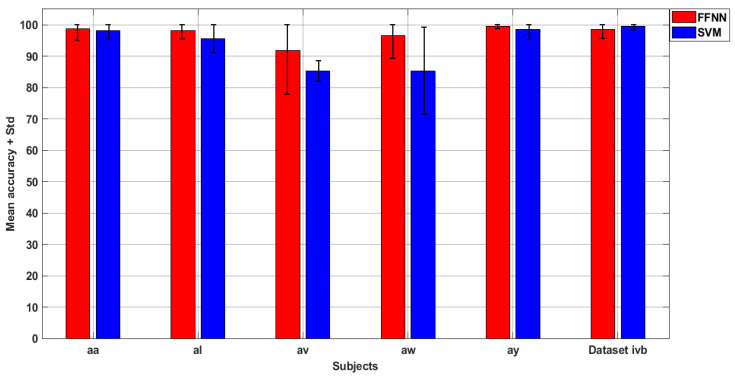
Results obtained with 10-fold 10 times.

To investigate the fallouts of classifier parameters on the proposed approach, we compared the classification accuracies for varying classifier parameters of all classifiers. Table 3 shows the averaged 10-fold accuracies of all classifiers with varying parameters for the 118-channel scheme using dataset IVa individual subjects and dataset IVb. For neural network (NN) classifiers, the number of hidden layer neurons was varied and its effect was observed accordingly. For SVM classifier, three different kernels namely radial basis function (RBF), linear kernel and the polynomial kernel were utilized, for DA classifier, linear, pseudo quadratic and pseudo linear kernels were adopted and their performance was evaluated for both datasets individually. The findings are as following:

1. The experimental results suggest that NN classifiers have no significant effect on average accuracy by varying the number of hidden neurons. For NN classifiers, the maximum mean classification accuracy was recorded for 40 neurons with 94.6% and 99.05% results for dataset IVa and IVb, respectively. For MNN classifier, the best case mean classification outcome was obtained for 30 neurons for dataset IVa with an accuracy of 91.2% and 40 neurons for dataset IVb resulting in 94.29% mean accuracy. Moving on to CFNN classifier, it is noted that each number of neurons yields 97% accuracy for dataset IVa and 99% outcomes for dataset IVb. Lastly, FFNN classifier turned out the maximum mean accuracy of 98.27% using dataset IVa and 99.52% using dataset IVb for 30 neurons each.
2. Amid the three kernels of the SVM classifier, it is observed that polynomial kernel is the best performant among others with the mean classification accuracy of 93.08% and 99.52% for datasets IVa and IVb, respectively. The linear and RBF kernels are ranked second and third accordingly with mean accuracies of 89.64% and 70.24% for dataset IVa and 96.19% and 83.33% for dataset IVb respectively.
3. In case of the DA classifier, we observe that the linear kernel is ranked highest as compared to pseudo-quadratic and pseudo-linear kernels. The average classification accuracy for linear kernel is 95.29% and 98.1% for dataset IVa and IVb each. Moving forward, the pseudo-linear kernel is ranked second with a mean classification accuracy of 93.29% and 95.1%, respectively, for datasets IVa and IVb. Last, the pseudo quadratic-kernel results in an average classification result of 92.63% and 92.24% accordingly for each dataset.
4. The thorough investigation of varying classifiers parameters suggests that polynomial and linear kernels are the best performers for SVM and DA classifiers respectively. Similarly, for NN, MNN, CFNN, and FFNN classifiers, 40 number of hidden neurons were chosen as the best parameter setting and these parameters are employed throughout this study.
5. Figure 7 shows the average accuracies of 10 times repeated 10-fold experiments for best (FFNN) and worst (SVM) case classifiers and each subject of dataset IVa and IVb. It is noted that the average results obtained for both classes results in slight variations of ±1.5%. In case of “av” subject with the FFNN and subject “aw” with the SVM, the variations are larger than 10%, which is due to the outliers caused by classifiers in some fold results but the mean results are more or less the same as calculated previously. The extensive experimentation results obtained confirms the robustness and stability of SDI features in estimating motor imagery tasks.

### 6.5. Results with Raw EEG and Noise-Free EEG Signals

We discussed earlier that EEG is a noninvasive mode of signal retrieval and it inherits noise artifacts while recording the data. In this section, a comparative analysis for MSPCA denoised and unprocessed (noisy) data is performed and validated if SDI feature is being affected by noise artifacts or not.

**Figure 8:**
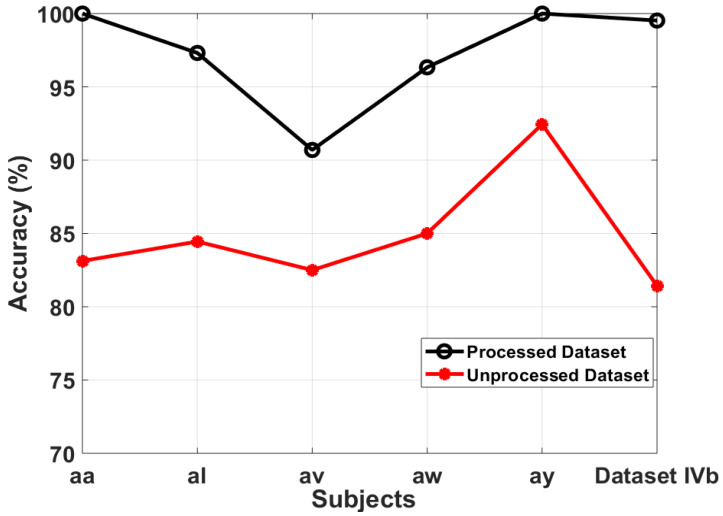
Comparison between denoised and noisy datasets.

Figure 8 shows the classification accuracy for MSPCA denoised and noisy data of dataset IVa and IVb. The classification results are calculated for best-case FFNN classifier. As observed from Figure 8, the classification accuracies for noisy data are 83.1%, 84.4%, 82.5%, 85%, 92.4%, and 81.4% for subjects “aa”, “al”, “av”, “aw”, “ay”, and dataset IVb, respectively. The average results are 85.5% and 81.4% for dataset IVa and IVb respectively. We observe a significant improvement in individual and average classification results after denoising the data. The results after denoising with MSPCA are 100%, 97.3%, 90.6%, 96.3%, 100% and 99.52% for subjects “aa”, “al”, “av”, “aw”, “ay”, and dataset IVb, respectively. The average accuracies for datasets IVa and IVb are 96.8% and 99.52%, respectively. By looking at the results obtained from two case scenarios, we observe an increase of 11.3% and 18.12% in accuracy for dataset IVa and IVb jointly. A similar trend of accuracy enhancement for denoised data was observed for other classifiers and hence it is concluded that the proposed SDI based feature extraction framework is robust against noise artifacts.

It is important to note that we have also checked numerous conventional methods including such band pass filters, temporal filtering, and spatial filtering for meticulous selection of a suitable strategy in the preprocessing module and identified that MSPCA produces the best findings for the proposed SDI feature extraction approach.

### 6.6. Classification Performance (%) with Dataset

This section deals with the experimental results of multiclass mental imagery dataset V. At first, the dataset was denoised with MSPCA and rearranged into individual trials with dimensions 512 × 32 (where 512 is the signal length and 32 is the number of channels) for each trial. We have rearranged the multiclass problem into 3 binary class experiments for each subject. The number of cases are given in Table 4. Here cases 1 to 3 are dedicated for participant 1 (P1), cases 4 to 6 corresponds to the participant 2 (P2). and cases 7 to 9 are formed for the participant 3 (P3). Next, the SDI feature is calculated for all trials and fed into six classifiers. The classification outcomes in terms of accuracies are given in Table 5.

It is observed from Table 5 that all classifiers achieved an average accuracy of above 90% for each subject. Moreover, the average individual classification accuracy for NN, MNN, CFNN and FFNN is above 95% which shows the effectiveness of NN classifiers in segregating mental imagery tasks. The best-case scenario was observed in for FFNN classifier with an average accuracy of 99.07%, 98.16%. and 98.38% for participants 1, 2, and 3, respectively. It should be noted that FFNN was the best performer for motor imagery tasks and now it again gives the best results for mental imagery dataset. The worst-case scenario was observed for SVM classifier with accuracies 91.84%, 90.36%, and 93.81%, respectively, for first participant, second participant, and third participant. As per the experimental results, it is concluded that NN classifiers, especially FFNN classifier is intelligent in estimating mental imagery tasks.

**Figure 9:**
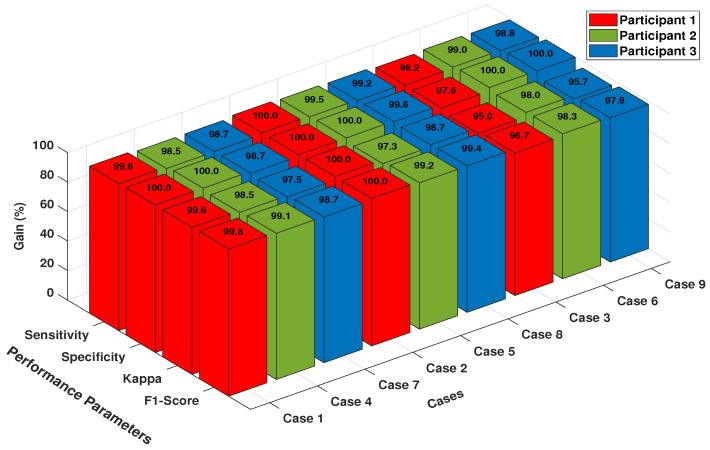
Performance parameters of FFNN classifier for Dataset V.

Figure 9 shows the classification performance of SDI feature for dataset V in terms of four performance parameters (Sensitivity, Specificity, Kappa, and F1-Score). The performance parameters are shown for the best classifier which is FFNN in our case. It can be inferred from Figure 9 that the sensitivity and specificity values for all cases in each subject are above 95% and in some cases, it is 100% which shows the greatness of FFNN classifier in predicting Class 1, Class 2, and Class 3 tasks. It can also be seen that the kappa and F1-measures are above 95% in all cases which depict the stabilization and unbiased nature of FFNN classifier. Overall it can be concluded that SDI features are not only specific for motor imagery tasks but equally essential and significant for mental imagery tasks as well.

### 6.7. CADMMI-SDI Application

**Figure 10:**
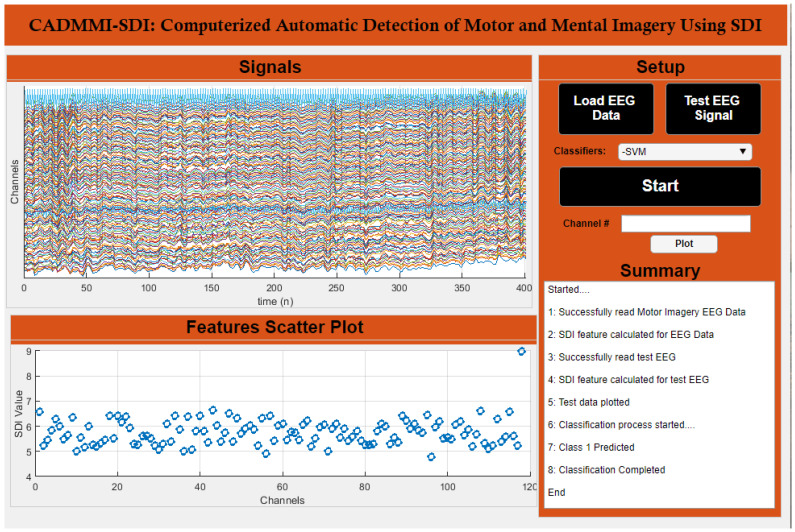
A display of CADMMI-SDI portraying all features and functionalities.

Apart from the theoretical analysis, we have developed a computerized automatic detection of motor and mental imagery using SDI (CADMMI-SDI) graphical user interface to assist physicians and laymen to utilize SDI method for their purpose without having to implement it their self. Table 6 presents the description of individual components present in the GUI while Figure 10 shows the detailed interface of our developed CADMMI-SDI. Some interesting features of the developed application are detailed in Table 6.

The demonstration of the GUI application can be seen in link https://www.youtube.com/watch?v=ugWbq4JUtuI. A copy of the GUI application is freely available and interested readers are suggested to write an email to corresponding author.

### 6.8. Computational Complexity of SDI Feature

**Figure 11:**
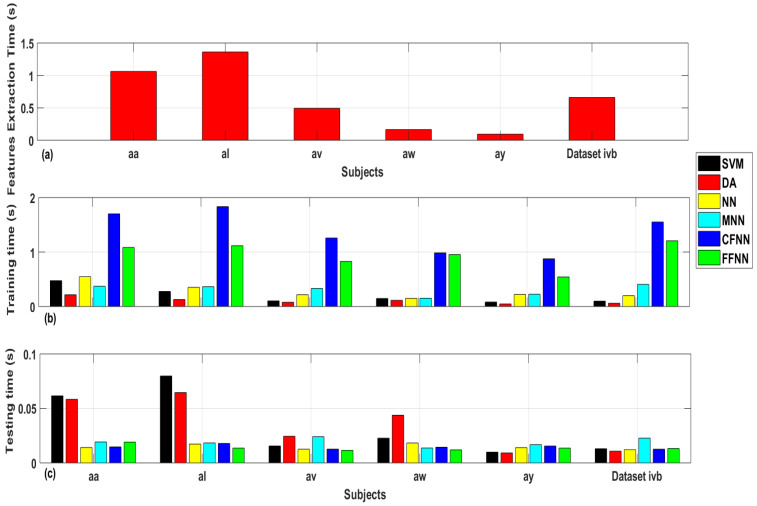
Bar plots representing time complexity: (**a**) Execution Time for SDI feature extraction method. (**b**) Training time. (**c**) Testing time.

Figure 11 shows the computational time for feature extraction, training and testing for all subjects and classifiers using the system specifications given in Section 5. First of all, Figure 11a presents the all trials feature extraction time for each subject of dataset IVa and dataset IVb. It can be seen that the highest feature extraction time of 1.36 s is taken by subject “al” followed by subject “aa” and dataset IVb with 1.06 s and 0.65 s, respectively. The average single-trial feature extraction time is calculated to be 0.85 milliseconds. Next, Figure 11b shows all trials training time for individual subjects and all classifiers. It is observed that CFNN classifier takes the highest training time for all subjects followed by FFNN classifier. The highest training time of 1.8 s, 1.75 s and 1.5 s was recorded for subjects “al”, “aa”, and dataset IVb, respectively, using CFNN classifier. The highest training time recorded for FFNN classifier is 1.2 s, 1.1 s and 1.08 s for dataset IVa, subject “al” and “aa”, respectively. The average single-trial training time for FFNN classifier is calculated to be 1.27 milliseconds. Last, Figure 11c shows all trials testing time for individual subjects and all classifiers. As noted, SVM classifier takes the highest testing time of 70 milliseconds and 60 milliseconds for subjects “al”, and “aa”, respectively. The time taken by FFNN classifier is minimum in most cases and the average single-trial training time is recorded to be 0.01 milliseconds. By accumulating the single trials computational times for FFNN classifier, it comes out to be 2.13 milliseconds which is very nominal as compared to other complex signal decomposition methods and it shows that besides noise robustness and classification accuracy, SDI features are computationally less complex and efficient and hence it can be employed in the production of practical BCI systems.

### 6.9. Performance Comparison with Other Literature

This section presents a comparative analysis of the proposed SDI framework with other recent state of art methods. Table 7 compares the classification accuracies for dataset IVa individual subjects and the best-case results are highlighted to make a fair comparison of other methods with the proposed approach. It can be seen from the table that subjects “aa” and “ay” attained 100% classification accuracy which is the highest among other methods. The results for subjects “al”,“av”, and “aw” are above 90% and very close to the best results achieved by other methods. Comparing the results of SDI feature method with our previous studies [23,24], it is worth noting that our current method outperforms the complex signal decomposition and modes selection-based methods. It can be noted from Table 7 that our method achieved the highest average classification accuracy of 97.54% with minimal heterogeneity. Moreover, there is a 24.04% maximum gain in accuracy comparing to other state of the art methods and hence it suffices that SDI feature extraction is not only efficient and non-complex but also robust in estimating motor imagery EEG signals and this is validated by a fair comparison with other widely acclaimed studies.

Table 8 shows the comparative results for multiclass dataset V. The outcomes are presented in terms of average classification accuracies and the highest case results are highlighted to make the best combination stand out. It is worth noting that the proposed SDI method outperformed all other methods in terms of individual subject results. It can be seen that the SDI method attained an average classification outcome of 99.07%, 98.16%, and 98.37% for participant 1, participant 2, and participant 3, respectively, and these are highest as compared to other methods. In terms of overall average results, the proposed SDI framework scored the highest 98.53% accuracy with a standard deviation of 0.387 that shows the consistency of overall results. Last, it is inferred from the comparison that SDI feature extraction method gains a minimum of 15.26% average classification accuracy, which is a significant improvement and it shows that the proposed method is not only useful for binary class motor imagery datasets but equally significant for multiclass mental imagery dataset as well.

Besides classification results, it is important to compare the complexity of other methods with SDI feature extraction method. As mentioned earlier in this study, our method has no signal decomposition, complex multidomain features extraction, or features selection procedures involved, which makes it computationally simple and less time-consuming. The studies in [22,23,24] use signal decomposition techniques that involves resolution of a time signal into different modes, then extraction of complex features and lastly selection of highly uncorrelated features. Such systems might be useful for the research analysis but they are not feasible to be adopted for practical BCI systems. Similarly, the studies [18,19,53] employs common spatial pattern (CSP)-based methods, which is another complex method for the analysis of EEG signals. The crux of the matter is whether we consider robustness, efficiency and complexity, the proposed SDI method outperforms all state-of-the-art methods in every aspect and gives us a feasible solution to be considered for the development of practical BCI systems.

### 6.10. Future Recommendations

In the present study, we utilized data with class labels, however, semisupervised learning or transductive learning methods are attracting attention these days. In future, researchers are encouraged to implement these methods for MI classification and information for these methods can be found in [58,59]. It is also worth mentioning that here, in the present study, we focused on at most three classes and presented the results in Table 5. However, for more number of classes readers should focus on more innovative strategies such as available in [60].

## 7. Conclusions

This study exploits the successive decomposition index (SDI) for the feature estimation of motor and mental imagery tasks. Three publicly available datasets namely dataset IVa, dataset IVb and dataset V from BCI competition III were utilized to attest the effectiveness of proposed method. Initially, the data was denoised with MSPCA and distributed into individual trials. Then, the SDI algorithm was used to calculate the feature corresponding to each trial and build a feature matrix for individual class instances. For the analysis purpose, a statistical test was performed that comprised mean, median, standard deviation, and Kruskal–Wallis nonparametric test for individual trials and it confirmed the efficacy of SDI as a potential feature. Moreover, a single evaluation metric named polygon area metric is employed to avoid looking into long tables. To validate the performance of the said method corresponding to the number of channels, four different channel selection criteria were tested and it confirmed that the 118-channel scheme has the leading results among other combinations. Furthermore, the classifier parameters were varied and a comparison between denoised and noisy data was performed to certify its effect on the classification performance of SDI feature. We also carried out a test for multiclass dataset V, and it was concluded that the proposed method is equally significant for the binary class as well as multiclass data. In the end, a computerized automated system CADMMI-SDI was developed for the practical realization of the proposed method. A comprehensive comparison of this study is made with other state of the art methods and it confirmed that the proposed method is robust, efficient, less complex and it can be utilized for the development of practical BCI systems.

## Figures and Tables

**Table 1:** Statistical analysis.

Participants	MI Tasks	Mean	Std	Median	KW *p* Values
“aa“	”Class 1 (RH)“	4.071	0.506	4.088	2.16 × 10^−19^
	”Class 2 (RF)”	4.633	0.175	4.626	
“al”	“Class 1 (RH)”	3.994	0.498	3.986	0.06112
	“Class 2 (RF)”	4.333	0.931	4.178	
“av”	“Class 1 (RH)”	3.655	0.516	3.698	3.27 × 10^−40^
	“Class 2 (RF)”	5.961	0.139	5.979	
“aw”	“Class 1 (RH)”	3.811	0.343	3.737	0.0001927
	“Class 2 (RF)”	3.966	0.323	3.925	
“ay”	“Class 1 (RH)”	5.766	0.535	5.725	5.81 × 10^−39^
	“Class 2 (RF)”	3.948	0.660	4.058	
“IVb”	“Class 1 (LH)”	3.716	0.440	3.734	4.09 × 10^−5^
	“Class 2 (RF)”	3.993	0.524	3.979	

**Table 2:** List of 3 channels automated criteria.

Subjects	Selected Channels
aa	CCP5, CP5, CP6
al	C3, FFC7, CCP3
av	FT9, P8, PPO8
aw	C4, CCP6, CP6
ay	CCP5, C3, CFC5
IVb	C3, CCP5, C4

**Table 3:** Classification (%) results for different parameters of the classifier.

“Classifiers”	“Variations in Parameters”	“aa”	“al”	“av”	“aw”	“ay”	“Dataset IVb”
“NN”	“5 Neurons”	97.61	93.30	77.78	83.67	95.43	99.52
	“10 Neurons”	95.00	91.05	86.67	90.67	97.33	99.05
	“20 Neurons”	97.61	98.20	87.22	89.33	98.23	97.62
	“30 Neurons”	97.61	97.33	76.94	93.00	99.24	98.57
	“40 Neurons”	99.41	95.49	85.97	93.00	99.10	99.05
“MNN”	“5 Neurons”	87.32	84.55	81.25	81.00	97.55	98.10
	“10 Neurons”	89.71	88.30	72.78	63.00	98.33	92.38
	“20 Neurons”	89.71	86.68	74.86	80.67	99.12	97.14
	“30 Neurons”	95.74	94.58	78.33	91.00	96.34	93.33
	“40 Neurons”	96.99	96.42	79.72	81.67	99.56	94.29
“CFNN”	“5 Neurons”	99.38	98.66	94.03	98.33	98.12	99.05
	“10 Neurons”	99.41	99.55	93.06	98.33	97.24	99.52
	“20 Neurons”	100.00	97.77	98.75	91.00	99.10	99.23
	“30 Neurons”	99.41	99.55	95.28	91.33	100	99.52
	“40 Neurons”	100.00	100.00	97.64	94.33	95.00	99.05
“FFNN”	“5 Neurons”	99.38	99.11	94.03	96.33	97.11	98.10
	“10 Neurons”	98.75	98.24	91.81	98.00	98.67	98.57
	“20 Neurons”	100.00	97.31	90.69	96.33	97.98	99.52
	“30 Neurons”	99.41	98.66	95.28	98.33	99.65	99.52
	“40 Neurons”	100	98.33	92.7	96.7	100	99.5
“SVM”	“RBF”	63.57	62.92	64.58	69.00	91.12	83.33
	“Linear”	94.08	88.38	85.42	84.67	95.65	96.19
	“Polynomial”	98.20	95.53	87.22	85.33	99.12	99.52
“DA”	“Linear”	98.82	99.09	86.53	94.67	97.33	98.10
	“Pseudo Quadratic”	94.12	97.81	84.58	86.67	100.00	93.24
	“Pseudo Linear”	98.82	99.09	85.42	84.00	99.12	95.10

**Table 4:** Different cases consider for SDI experimental work by employing dataset V.

**Case 1:**	“Class 1 (LH)” vs. “Class 2 (RH)”	**Case 2:**	“Class 1 (LH)” vs. “Class 3 (RW)”	**Case 3:**	Class 2 (RH) vs. “Class 3 (RW)”
**Case 4:**	“Class 1 (LH)“ vs. ”Class 2 (RH)“	**Case 5:**	”Class 1 (LH)“ vs. ”Class 3 (RW)“	**Case 6:**	”Class 2 (RH)“ vs. ”Class 3 (RW)“
**Case 7:**	”Class 1 (LH)“ vs. ”Class 2 (RH)“	**Case 8:**	”Class 1 (LH)“ vs. ”Class 3 (RW)“	**Case 9:**	”Class 2 (RH)“ vs. ”Class 3 (RW)“

**Table 5:** Classification accuracies (%) obtained with different cases by employing dataset V.

Classifiers	Cases	“P1”	“P2”	“P3”
“NN”	Case 1	100.00	98.22	96.67
	Case 2	93.21	97.98	97.44
	Case 3	100.00	97.12	98.43
	**Average**	**97.74**	**97.77**	**97.51**
“MNN”	Case 1	100.00	99.88	93.08
	Case 2	98.43	95.16	98.30
	Case 3	93.21	90.34	94.12
	**Average**	**97.21**	**95.13**	**95.16**
“CFNN”	Case 1	98.44	99.12	97.12
	Case 2	98.49	99.12	98.45
	Case 3	96.34	99.34	96.34
	**Average**	**97.76**	**99.19**	**97.30**
“FFNN”	Case 1	99.12	98.24	99.89
	Case 2	100.00	99.13	97.12
	Case 3	98.09	97.12	98.12
	**Average**	**99.07**	**98.16**	**98.38**
“SVM”	Case 1	95.23	94.32	99.12
	Case 2	94.74	95.23	94.98
	Case 3	85.54	81.52	87.34
	**Average**	**91.84**	**90.36**	**93.81**
“DA”	Case 1	93.45	94.55	93.19
	Case 2	94.14	96.34	95.83
	Case 3	88.32	91.78	89.15
	**Average**	**91.97**	**94.22**	**92.72**

**Table 6:** CADMMI-SDI application.

Application Components	Description
Load EEG Data	Load Sample EEG data for a specified destination. The file type must be *.csv or *.xlsx
Test EEG Signal	Load test data from a specific folder. The file format should be *.csv or *.xlsx
Classifiers	Choose a classifier by drop-down selection.
Start	A key to initiate/start the process
Channel #	Input desired number of channels and press “Plot” to display. The channel number should be separated by a comma
Summary	Text section to demonstrate the specifics of the process underway
Signals	2D plot window to display EEG signals
Features Scatter Plot	2D plot window to display SDI feature corresponding to each channel

**Table 7:** Performance comparison of motor imagery EEG signals in terms of classification accuracy (%) with other literature.

Methods By	Suggested Methods	Classification Accuracy (%)						
		“aa”	“al”	“av”	“aw”	“ay”	“Avg.”	“Std.”
**Our Present Work**	**“Successive decomposition index tested with feedforward neural network”/the proposed**	**100**	98.3	92.7	96.7	**100**	**97.5**	2.7
our previous work in 2019 [24]	“Multivariate empirical wavelet transform tested with least-square support vector machines”	95	95	95	**100**	**100**	97	2.7
our previous work in 2019 [23]	“Empirical wavelet transform tested with least-square support vector machines”/our last work	94.5	91.7	97.2	95.6	97	95.2	2.3
work by “Wu” et al. in 2008 [48]	“Iterative spatio-spectral patterns learning”	93.6	**100**	79.3	99.6	98.6	94.2	8.7
work by “Kevric” et al. in 2017 [22]	“Wavelet packet decomposition tested with K nearest neighbors”	96	92.3	88.9	95.4	91.4	92.8	2.9
work by “Siuly” et al. in 2011 [49]	“Clustering tested with least-square support vector machines“	92.6	84.9	90.8	86.5	86.7	88.3	3.2
work by ”Song“ et al. in 2007 [50]	“Common spatial pattern tested with support vector machines”	87.4	97.4	69.7	96.8	88.6	87.9	11.2
work by “Lu” et al. in 2010 [19]	“Regularized common spatial pattern tested with aggregation“	76.8	98.2	74.5	92.2	77	83.7	10.7
work by ”Zhang“ et al. in 2013 [51]	“Z-score tested with linear discriminant analysis”	77.7	**100**	68.4	99.6	59.9	81.1	18.1
work by “Lotte“ et al. in 2010 [18]	”Regularized common spatial pattern with selected subjects“.	70.5	96.4	53.5	71.9	75.4	73.6	15.3
	”Common spatial pattern with Tikhonov regularization“.	71.4	96.4	63.3	71.9	86.9	77.9	13.4
	”Common spatial pattern with weighted Tikhonov regularization“.	69.6	98.2	54.6	71.9	85.3	75.9	16.6
	”Spatially regularization common spatial pattern“.	72.3	96.4	60.2	77.7	86.5	78.6	13.8
work by ”Yong“ et al. in 2008 [52]	”Sparse spatial filter optimization“	57.5	86.9	54.4	84.4	84.3	73.5	16

**Table 8:** Performance comparison of mental imagery EEG signals in terms of classification accuracy (%) with other literature.

Methods By	Suggested Methods	Classification Accuracy (%)				
		“P1”	“P2”	“P3”	“Avg.”	“Std.”
**Our Present Work**	**“Successive decomposition index tested with feedforward neural network”/The proposed**	88.9	**94.1**	**92.4**	**91.8**	**2.6**
work by “Siuly” et al. in 2017 [54]	“Principal component analysis employed with random forest”	**91.8**	75.2	82.8	83.3	8.3
research by “Lin” et al. in 2009 [55]	“Modified partical swarm optimization employed with neural networks”	78.3	75.2	56.5	69.9	11.81
work by “Siuly” et al. in 2011 [49]	“Clustering employed with least-square support vector machines”	68.2	64.8	52.1	61.7	8.5
experiments by “Sun” et al. in 2008 [56]	“Selection of electrodes with the help of Ensemble method”	68.7	56.4	44.8	56.7	11.9
work by “Sun” et al. in 2007 [57]	“Ensemble Methods”	70.6	48.9	40.9	53.4	15.4
work by “Sun” et al. in 2009 [53]	“Automated common spatial method”	67.7	68.1	59.6	65.1	4.82
